# Meta-analysis: the diagnostic accuracy of critical flicker frequency in minimal hepatic encephalopathy

**DOI:** 10.1111/apt.12199

**Published:** 2013-01-07

**Authors:** F J Torlot, M J W McPhail, S D Taylor-Robinson

**Affiliations:** Hepatology & Gastroenterology Section, Division of Diabetes, Endocrinology & Metabolism, Department of Medicine, St Mary's Hospital Campus, Imperial College LondonLondon, UK

## Abstract

**Background:**

Minimal hepatic encephalopathy (MHE) reduces quality of life, increases the risk of road traffic incidents and predicts progression to overt hepatic encephalopathy and death. Current psychometry-based diagnostic methods are effective, but time-consuming and a universal ‘gold standard’ test has yet to be agreed upon. Critical Flicker Frequency (CFF) is a proposed language-independent diagnostic tool for MHE, but its accuracy has yet to be confirmed.

**Aim:**

To assess the diagnostic accuracy of CFF for MHE by performing a systematic review and meta-analysis of all studies, which report on the diagnostic accuracy of this test.

**Methods:**

A systematic literature search was performed to locate all publications reporting on the diagnostic accuracy of CFF for MHE. Data were extracted from 2 × 2 tables or calculated from reported accuracy data. Collated data were meta-analysed for sensitivity, specificity, diagnostic odds ratio (DOR) and summary receiver operator curve (sROC) analysis. Prespecified subgroup analysis and meta-regression were also performed.

**Results:**

Nine studies with data for 622 patients were included. Summary sensitivity was 61% (95% CI: 55–67), specificity 79% (95% CI: 75–83) and DOR 10.9 (95% CI: 4.2–28.3). A symmetrical sROC gave an area under the receiver operator curve of 0.84 (SE = 0.06). The heterogeneity of the DOR was 74%.

**Conclusions:**

Critical Flicker Frequency has a high specificity and moderate sensitivity for diagnosing minimal hepatic encephalopathy. Given the advantages of language independence and being both simple to perform and interpret, we suggest the use of critical flicker frequency as an adjunct (but not replacement) to psychometric testing.

## Introduction

Minimal hepatic encephalopathy (MHE) is an important clinical variant of hepatic encephalopathy (HE), which occurs in up to 60–70% of patients with cirrhosis.^1^, ^2^ The condition comprises a cognitive impairment, observed in patients with cirrhosis who have no clinical evidence of overt hepatic encephalopathy (OHE).^3^ It is associated with an increased incidence of road traffic accidents,^4^–^7^ reduced quality of life and it affects the ability to perform tasks of daily living.^8^, ^9^ It has also been shown to increase the risk of progressing to OHE and inversely correlates with survival in some studies.^10^–^12^.

Treatment for MHE can improve psychometric performance and health-related quality of life.^13^–^17^ It is therefore clinically useful that MHE is diagnosed effectively in patients with this condition. A quick, accurate, objective, cost-effective and well-validated diagnostic test is an unmet clinical need and would simplify the early management algorithm for this condition.

Minimal hepatic encephalopathy is not routinely tested, even in specialised cirrhosis clinics. A survey among members of the American Association for the Study of Liver Diseases (AASLD) showed that 72% tested less than half their patients for MHE, despite 84% acknowledging that MHE is a significant problem. Furthermore, 85% said that if clinical staff could perform a quick, accurate test, this would increase the likelihood that people would test for MHE.^18^

There are many diagnostic tests for MHE, but no universal ‘gold standard’ test. The Expert Working Group in 1998 suggested that the psychometric hepatic encephalopathy score (PHES) should be considered the gold standard test.^19^ PHES is a selection of five psychometric test batteries that has been validated in Italian,^20^ German^21^, ^22^ and Spanish^23^ cohorts. However, performing this battery can be time consuming and prone to bias from disturbance, mood and interaction with the tester.

Computer-assisted tests have also been used to diagnose MHE and include the Inhibitory Control Test (ICT),^24^, ^25^ the cognitive drug research system (CDRS),^26^ the continuous reaction time test (CRT)^27^, ^28^ and Critical Flicker Frequency (CFF).^29^ The CFF has the purported advantages of not being dependent on language, verbal fluency, numeracy or numerics, and therefore studies into its use have been performed in the United States, Europe and Asia.

The CFF was devised originally as an ophthalmological test used to measure visual acuity and to screen for optic nerve lesions.^30^ This test measures the frequency at which the patient perceives that a fused/single light becomes a flickering light. The device causes a stepwise decrease in frequency from 60 to 25 Hz. This is done multiple times (usually 8–10) to allow calculation of the mean and standard deviation. It may also be performed in reverse, where the patient determines the frequency at which a flickering light becomes continuous or ‘fused’. This test has the advantage of being able to be carried out by clinical personnel using a portable device with limited running costs.^31^

The CFF has been in limited clinical and research use for a decade, but its diagnostic accuracy has never been subjected to quantitative review. We therefore performed a systematic review and meta-analysis of CFF to fully assess its diagnostic accuracy in detecting MHE and to guide future implementation.

## Materials And Methods

### Search methodology

We searched MEDLINE and EMBASE using OvidSP for articles between January 1948 and November 2012, which reported on the diagnostic accuracy of CFF for MHE. The search terms used were ‘Critical Flicker Frequency’ OR ‘CFF’ in conjunction (AND) with ‘Diagnostic Accuracy’ OR MeSH term ‘Diagnostic techniques and procedures’ OR ‘Sensitivity’ OR ‘Specificity’. Inclusion criteria were adult studies reporting the diagnostic accuracy for CFF in patients with cirrhosis and/or portosystemic bypass. Paediatric studies, studies not related to MHE in the context of cirrhosis or portosystemic bypass, those which did not refer to a gold standard and those not in the English language were excluded. We also obtained primary sources from tracking references from hand searches in review papers and original articles. Only original data were used in the meta-analysis.

### Data extraction

Test performance data were extracted as a 2 × 2 table of true negative, true positive, false positive and false negative values directly from tabulated results. If these were not available, they were calculated from reported sensitivity, specificity, positive predictive value and/or negative predictive values; if this were not possible, the authors were contacted for more detailed data; and if this was not possible or there was doubt over the 2 × 2 calculation, the study was excluded from subsequent analysis.

### Assessment of study quality

Studies meeting the above criteria were quality assessed using positive scoring in a modified 23-point Standards for the Reporting of Diagnostic Accuracy Studies (STARD) pro forma.^32^, ^33^ We modified this scoring system from 25 to 23 as two points were not relevant to this study (points 18 and 20). Two readers (FT and MM) independently assessed all included studies according to the prearranged pro forma. An open discussion was then held to determine any disagreement between the readers. Studies were then segregated into ‘low’ or ‘high’ quality depending on whether they met < or > than 50% of the study quality pro forma (Low <12/23, High ≥12/23).

### Data analysis

The DerSimonian-Laird random effects method was used to produce summary estimates of sensitivity, specificity, likelihood ratios (LR) and diagnostic odds ratio (DOR). Confidence intervals for sensitivity and specificity were calculated using F-distribution method for the binomial proportion.^34^ The summary receiver operator curve (sROC) was used to graphically determine performance following testing for correlation between sensitivity and specificity [as logit true positive rate (TPR) vs. logit false positive rate (FPR)] to explore for threshold effects and subsequent assessment for constant DOR using the Moses-Sharpiro-Littenburg model.^35^ Symmetrical or asymmetrical sROC were used depending on whether the DOR is constant. Heterogeneity was investigated using pre-planned subgroup analysis and calculated by the *I*^2^ method.^36^ Pre-planned subgroups were defined according to: study quality (low or high with 50% quality pro forma cut-off used), type of gold standard test (PHES or non-PHES), study location (Europe or non-European), CFF cut-off (≤38 or ≥39), whether the study was published in early era or late era (median study year from extracted studies was used as the cut-off), number of patients in the respective studies (<50 subjects or ≥50 subjects)(50 = median) and the aetiology of the MHE (cirrhosis or bypass/shunts).

A funnel plot and effective sample size (ESS) regression analysis (the logarithm of the DOR plotted against 1/√ESS) was used to investigate publication bias. 1/√ESS is proportional to root (1/n1 + 1/n2) where n1 = number diseased and n2 number not diseased.^37^ Data analyses were performed using the freeware Meta-Disc version 1.4 (Universidad Complutense, Madrid, Spain).^38^

## Results

**Figure 1:**
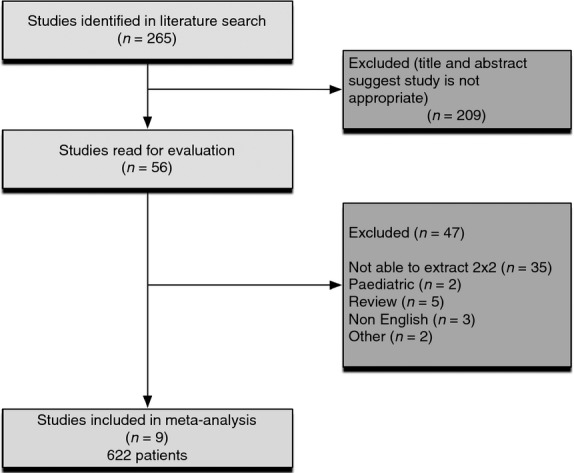
Flow diagram of studies identified in the systematic review.

The search strategy identified 265 studies, 209 were excluded, based on title and abstract, while the remaining 56 were read and evaluated. Forty seven were further excluded, based on prestated criteria leaving 9, which were included in the final meta-analysis^29^, ^39^–^46^ (Figure 1)

Data from 622 patients were available. Four of nine studies were published in the early era (2002–2008) and five were published in the late era (2009–2011). Five studies were deemed to be of low quality (<12/23) and the other four studies were deemed to be of high quality (≥12/23), according to the predescribed pro forma.^32^, ^33^ Four studies used PHES as their reference test, whereas five studies used non-PHES as reference tests. Four studies were conducted in Europe, whereas five were performed elsewhere. Six studies used a CFF cut-off value of ≤38 Hz to distinguish an abnormal test, whereas the other three used a cut-off value of ≥39 Hz. The median (range) number of participants was 50 (31–114). Four studies had less than 50 patients and five studies had 50 or more patients.

We grouped studies into MHE caused by cirrhosis and MHE caused by bypass/shunting. Cirrhosis was the cause of the MHE in seven studies, while bypass/shunting [secondary to extra-hepatic portal venous obstruction (EHPVO)] was the cause in the other two.

Finally, we were unable to investigate different aetiologies of cirrhosis (such as alcohol or viral hepatitis) in our meta-analysis, as it was not possible to extract separate 2 × 2 tables for these aetiologies from the studies.

### Sensitivity, specificity and diagnostic odds ratio

**Figure 2:**
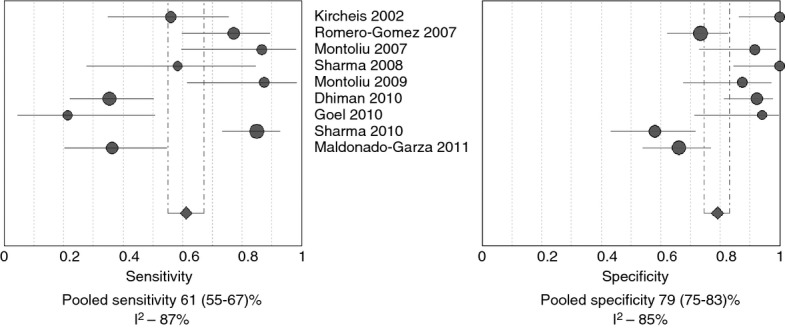
Forest plots for sensitivity and specificity for all nine studies.

The pooled sensitivity for all nine studies included in the final meta-analysis was 61% (95% CI: 55–67%), pooled specificity was 79% (95% CI: 75–83%), pooled positive LR was 3.5 (95% CI: 2.0–6.1), pooled negative LR was 0.46 (95% CI: 0.31–0.68) and the pooled DOR was 10.9 (95% CI: 4.2–28.3). (Table 1, Figure 2, Figures S1 and S2).

**Table 1:** Characteristics of the nine studies included in the meta-analysis

Study name	Sensitivity (95% CI)/%	Specificity (95% CI)/%	Positive LR (95% CI)	Negative LR (95% CI)	DOR (95% CI)	TP	FP	FN	TN	Reference test	STARD score
Kircheis 2002^29^	56 (35–76)	100 (86–100)	29.0 (1.8–461.1)	0.45 (0.29–0.70)	64.3 (3.5–1173.2)	14	0	11	25	A	14
Romero-Gomez 2007^39^	77 (60–90)	73 (62–83)	2.9 (1.9–4.4)	0.31 (0.17–0.58)	9.3 (3.7–23.7)	27	21	8	58	PHES	14
Montoliu 2007^30^	87 (60–98)	92 (73–99)	10.4 (2.7–39.8)	0.15 (0.04 –0.53)	71.5 (9.0–570.3)	13	2	2	22	PHES	6
Sharma 2008^41^	58 (28–85)	100 (85–100)	26.5 (1.6–428.1)	0.43 (0.23–0.82)	61.4 (3.0–1245.8)	7	0	5	22	B	11
Montoliu 2009^42^	88 (62–98)	88 (68–97)	7.0 (2.4–20.5)	0.14 (0.04–0.53)	49.0 (7.2–331.8)	14	3	2	21	PHES	7
Dhiman 2010^43^	35 (22–51)	92 (82–98)	4.6 (1.7–12.7)	0.70 (0.56–0.88)	6.6 (2.0–21.4)	17	4	31	48	C	13
Goel 2010^44^	21 (5–51)	94 (71–100)	3.6 (0.4–31.3)	0.84 (0.62–1.13)	4.4 (0.4–47.6)	3	1	11	16	D	6
Sharma 2010^45^	85 (73–93)	58 (43–72)	2.0 (1.4–2.9)	0.26 (0.14–0.49)	7.8 (3.2–19.3)	51	21	9	29	E	12
Maldonado-Garza 2011^46^	36 (20–55)	66 (54–77)	1.1 (0.6–1.9)	0.96 (0.71–1.31)	1.1 (0.5–2.7)	12	24	21	47	PHES	8
Pooled	61 (55–67)	79 (75–83)	3.5 (2.0–6.1)	0.46 (0.31–0.68)	10.9 (4.2–28.3)						

A – Computerised psychometry.

B – NCT A and B or FCT A and B, and/or abnormal P300 ERP.

C – NCT-A, FCT-A, SDT, DST, LTT for time and for error.

D – NCT A and B, FCT A and B, WAIS-P tests (PC, DS, PA, OA, BD).

E – NCT A and B or FCT A and B, and abnormal P300 ERP.

**Figure 3:**
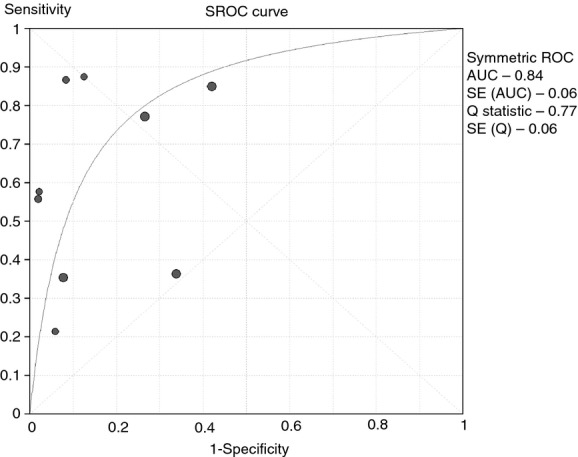
Symmetrical Summary Receiver Operator Curve (sROC) for all nine studies.

The Spearman correlation coefficient for logit (TPR) vs. logit (FPR) was 0.38 (*P* = 0.31), indicating a nonsignificant correlation between the TPR and FPR. The Moses-Shapiro-Littenberg method showed that the DOR was constant [b = −0.018 (*P* = 0.95)]. A symmetrical sROC was therefore the most appropriate representation of the diagnostic accuracy (Figure 3). It depicted an area under the receiver operator curve (AUROC) (SE) of 0.84 (0.06) and with a *Q* statistic (SE) of 0.77 (0.06).

### Heterogeneity analysis & subgroup analysis

Heterogeneity was detected in all pooled indices. For all nine studies, the heterogeneity (*I*^2^) was 87% (sensitivity), 85% (specificity), 72% (positive LR), 85% (negative LR), 74% (DOR).

Subgroup analysis was performed to assess differences in heterogeneity and diagnostic accuracy between the prespecified groups (Table 2). The heterogeneity of the DOR was lower in the high-quality studies (*I*^2^, 0% vs. 85%). There was less heterogeneity in studies, which used a non-PHES gold standard test (*I*^2^, 5% vs. 88%). We also found that there was less heterogeneity in the European studies, compared with non-European studies (*I*^2^, 45% vs. 72%). There was lower heterogeneity in the smaller studies (*I*^2^, 17% vs. 77%), in the studies with a CFF cut-off ≥39 (*I*^2^, 15% vs. 82%) and in the studies published in the early era (*I*^2^, 40% vs. 78%).

**Table 2:** Assessment of diagnostic accuracy and heterogeneity in subgroup analysis

Sub groups	No. of studies	Pooled sensitivity (95% CI)/%	Pooled specificity (95% CI)/%	Pooled positive LR (95% CI)	Pooled negative LR (95% CI)	Pooled DOR (95% CI)	*I*^2^ (%) DOR
All studies	9	61 (55–67)	79 (75–83)	3.5 (2.0–6.1)	0.46 (0.31–0.68)	10.9 (4.2–28.3)	74
Era							
Early (2002–2008)	4	70 (59–80)	85 (78–90)	8.8 (2.0–38.2)	0.37 (0.26–0.54)	26.6(7.3–97.4)	40
Late (2009–2011)	5	57 (49–64)	75 (69–81)	2.6 (1.4–4.9)	0.58 (0.37–0.91)	5.9 (1.8–19.4)	78
Quality assessment							
Low	5	54 (44–65)	81 (74–87)	5.0 (1.3–18.8)	0.47 (0.25–0.91)	14.1 (1.7–117.4)	85
High	4	65 (57–72)	78(71–83)	2.9(1.7–4.9)	0.42(0.24–0.73)	8.7(5.0–15.1)	0
Gold standard							
Non-PHES	5	58(50–66)	84(78–90)	4.9(1.7–14.3)	0.53(0.35–0.79)	8.8 (4.4–17.6)	5
PHES	4	67 (57–76)	75 (68–81)	3.3 (1.4–8.0)	0.31 (0.10–1.00)	11.7 (1.8–74.5)	88
Location							
Non-Europe	5	54 (46–62)	76 (70–82)	2.4 (1.2–4.6)	0.63 (0.44–0.91)	5.1 (1.6–15.9)	72
Europe	4	75 (65–83)	83 (76–89)	6.3 (2.3–17.2)	0.29 (0.16–0.51)	27.3 (8.2–91.2)	45
No. of patients							
<50	4	65 (51–77)	93 (86–97)	8.1 (3.8–17.1)	0.32 (0.10–1.08)	32.5 (9.3–113.3)	17
≥50	5	60 (53–67)	75 (69–80)	2.4 (1.4–4.0)	0.51 (0.32–0.80)	6.0 (2.1–17.2)	77
Cut-off							
≤38	6	57 (49–64)	80 (75–85)	4.0 (1.9–8.5)	0.44 (0.25–0.75)	12.0 (3.1–45.6)	82
≥39	3	69(59–78)	76(66–84)	4.1(0.8–20.6)	0.48(0.21–1.06)	9.1 (3.2–25.6)	15
Aetiology of MHE							
Bypass	2	39 (20–59)	97(87–100)	8.2 (1.1–59.8)	0.63 (0.29–1.37)	13.9 (1.0–186.4)	46
Cirrhosis	7	64 (57–70)	77 (72–81)	3.2 (1.9–5.6)	0.40 (0.25–0.67)	10.6 (3.7–30.5)	79

Critical Flicker Frequency was more diagnostically accurate in the European studies, DOR = 27.3 (95% CI: 8.2–91.2) compared with a DOR = 5.1 (95% CI: 1.6–15.9) for non-European studies. CFF was also more accurate in diagnosing MHE in studies performed in the early era DOR = 26.6 (95% CI: 7.3–97.4) compared with the late era DOR = 5.9 (95% CI: 1.8–19.4). Smaller studies (<50 patients) had a greater DOR = 32.5 (95% CI: 9.3–113.3), compared with the bigger studies (≥50 patients) DOR = 6.0 (95% CI: 2.1–17.2). There was little difference in the DOR among the Quality (low/high), cut-off (≤38/≥39) aetiology (bypass/cirrhosis) and the gold standard (non-PHES/PHES) subgroups. To assess if there was any significant correlation between any of these co-variables and the diagnostic accuracy of CFF (DOR), meta-regression analysis was performed.

### Meta-regression & Publication bias

There was no significant correlation between any of the covariates and the DOR in the univariate meta-regression analysis (Table 3). Owing to only nine studies being included in this meta-analysis, the power of multivariate meta-regression is low and thus this limits the overall value of meta-regression in this meta-analysis.

**Table 3:** Results of univariate meta-regression analysis of diagnostic odds ratio

Co-variables	*P-*value	RDOR	95% CI
Era (2002–2008)/(2009–2011)	0.15	0.19	(0.02–2.23)
Quality (low/high)	0.92	0.88	(0.05–15.11)
Gold Standard (non-PHES/PHES)	0.94	0.91	(0.04–21.29)
Location (non-Europe/Europe)	0.09	6.65	(0.70–63.62)
Number of patients (<50/≥50)	0.11	0.17	(0.02–1.77)
Cut-off (≤38/≥39)	0.95	0.92	(0.04–19.06)
Aetiology (Bypass/Cirrhosis)	0.90	0.77	(0.01–81.55)

RDORm, relative DOR.

**Figure 4:**
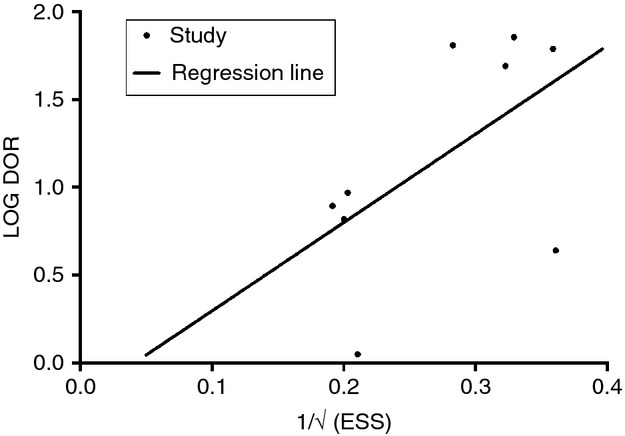
Deeks' funnel plot.

No significant publication bias was found in our study sample, as the linear regression analysis indicates *P* = 0.11 (Figure 4).

## Discussion

The importance of MHE as a complication of cirrhosis or portosystemic bypass has only been recognised in the last decade and its profound effect on patients with cirrhosis should not be underestimated. Neither, unfortunately, should clinicians' poor understanding of MHE and hence their reluctance to test for its presence.^18^ Added to this, there are a number of diagnostic options, but many clinicians are confused as to which to choose. In the absence of other easily implementable clinical alternatives, the PHES battery has been suggested as the current gold standard internationally for MHE diagnosis,^19^ but patient numeracy, literacy or language skills may affect the result.^47^ CFF has been proposed as being unaffected by these issues, but its diagnostic accuracy has never been analysed quantitatively before.

There are a number of broad themes that emerged from the meta-analysis. CFF only had a moderate pooled sensitivity of 61% (95% CI: 55–67), but a good specificity of 79% (95% CI: 75–83). The symmetrical sROC curve had an AUROC of 0.84, indicating that CFF was effective in discriminating patients with MHE from those without MHE and therefore has potential as a screening tool, either to be used prior to current psychometrics tools, or to be used alongside these tests, but not as a replacement for them given the risk of false negative results.

We used subgroup analysis to compare CFF diagnostic accuracy in MHE caused by portosystemic bypass/shunting (DOR = 13.9), compared with MHE caused by cirrhosis (DOR = 10.6). Further analysis shows that CFF has high specificity of 97% (95% CI: 87–100) for MHE in patients with bypass/shunting (secondary to EHPVO), but a low sensitivity of 39% (95% CI: 20–59). Further studies are needed to inform on whether CFF is effective for diagnosing MHE caused by bypass/shunting, and whether the pathogenesis of MHE in this condition predetermines the best diagnostic modality to choose. It may be that the neurophysiological impairment of the high ammonia states in portosystemic bypass/shunting compared the potentially lower levels of hyperammonaemia, but higher rates of inflammation in patients with cirrhosis affect the performance of CFF in these groups.^48^, ^49^

We found that the studies published in the early era had a higher DOR than studies published in the late era (DOR: 26.6 vs. 5.9). Although this difference was not significant on univariate meta-regression, it does question whether the high DOR seen in early studies^29^ might represent an overestimation of diagnostic accuracy, particularly when the test is applied to heterogeneous patient cohorts. We also found that the diagnostic accuracy was higher in studies published in Europe (DOR: 27.3 vs 5.1). Again, this showed no significance on meta-regression analysis, but may represent an early indicator that CFF may be more accurate in European patients. It remains unclear why this objective, language-independent test would perform better in these subgroups, but it is an important observation and does have implications on the role of CFF as a worldwide screening tool for MHE, particularly when the sensitivity is only 54% in the non-European, compared with 75% in the European subgroups.

We explored the possibility that early studies outperformed later studies due to the problem of publication bias, and we acknowledge that it can be a significant problem with diagnostic accuracy meta-analysis, but we found no statistical evidence of this across our study sample. We are also aware that four of the five non-European studies were published in India^41^, ^43^–^45^ and two of them by the same centre. Further studies are regarding the effect of location and race on this diagnostic test, particularly in non-European countries outside of India.

The considerable amount of heterogeneity detected between the studies suggests a need for caution when pooling the diagnostic accuracy measures together. We used subgroup analysis to assess the heterogeneity in prespecified groups, and unsurprisingly found that it was reduced in the ‘high quality’ studies. The implicit and explicit causes of threshold effect were assessed as another cause of heterogeneity. Most studies used either 38 Hz or 39 Hz as the flicker frequency cut-off value to discriminate between patients with or without MHE; when this was assessed in the meta-regression analysis, there was no correlation between this value and the DOR. We further assessed the implicit causes of threshold effect by calculating the Spearman correlation, which was 0.38 (*P* = 0.31). While there was no statistical evidence of a threshold effect, a summary ROC (sROC) remains a useful composite measure of the diagnostic accuracy of CFF.^50^ The Moses model showed the DOR to be constant, so we investigated further using a symmetrical sROC confirming the good overall accuracy of CFF.^35^

One limitation of this meta-analysis is that the nine studies included had to compare CFF to a reference test. This provides two problems: first, the reference test is not the same for all nine studies; and second, the diagnostic accuracy of this test may be less than 100%. Within the subgroup analysis, we looked at studies that compared CFF with PHES (the current suggested diagnostic gold standard) and studies that referenced to another non-PHES test (Table 1). Attempts were made to assess differences in heterogeneity and diagnostic accuracy between these two groups. The diagnostic accuracy for CFF was slightly higher in the PHES subgroup (DOR: 11.7 vs. 8.8), but also significantly more heterogeneous (DOR: 88% vs. 5%). Although there is some difference in diagnostic accuracy, the meta-regression analysis showed no significance. We can, therefore, cautiously conclude from this meta-analysis that CFF seems to perform comparably to PHES and non-PHES diagnosed MHE. The increase in heterogeneity observed may thus be explained to some extent.

One further limitation common to all diagnostic meta-analyses is the lack of clarity, quality and standardisation in diagnostic studies' methodology. Studies were assessed for quality using STARD pro forma to quantify the methodology of the study design. We had to exclude two studies at a late stage due to ambiguity between the raw data and the diagnostic accuracy data, which could not be resolved with the authors.^51^, ^52^ Furthermore, it should be noted that Maldonado-Garza and colleagues' study, for example, was not intended to be a diagnostic study, yet included sufficient data to enable its inclusion in this meta-analysis. We would encourage any further studies designed to assess the diagnostic accuracy of CFF to follow either the STARD or PRISMA checklist.

Despite the limitations, we know that CFF is a simple, affordable test, which is easy to perform. It is thus realistic for departments specialising in the management of patients with cirrhosis or portosystemic bypass/shunting, who are at high risk of MHE, to buy and use this device. The test does not require specialist personnel to conduct and is extremely well tolerated and easily understood by the patients within the studies. The only limitation of its use in the nine studies was in Romero-Gomez and colleagues study where nine patients and three controls could not perform the test due to visual impairment or inability to understand the fundamentals involved.^39^

Key to the management algorithm of MHE is a correct, early diagnosis, prompting early effective treatment.^13^–^17^ Many of the diagnostic tests available at present are time consuming and require trained personnel to perform them. Furthermore, the lack of universal consensus on which diagnostic methods and strategies should be implemented heightens the problem of under-diagnosis and as demonstrated, no uniformly implemented international gold standard diagnostic testing criteria for MHE exist. This meta-analysis has shown that CFF is a diagnostically accurate test, which could be used as an adjunct to conventional psychometric test batteries, such as PHES, but could only become a replacement screening test if further studies show an improvement in sensitivity.

## Authorship

*Guarantor of the article*: S. D. Taylor-Robinson

*Author contributions*: All authors contributed to the design of the study and writing of the manuscript. FT and MJM undertook the research and performed the analyses. All authors approved the final version of the manuscript.

## SUPPORTING INFORMATION

**Figure S1.** Forest plot for positive and negative likelihood ratios for the nine studies.

**Figure S2.** Forest plot for diagnostic odds ratio for the nine studies.
