# Supplementary figures and images for: Meta-analysis: the diagnostic accuracy of critical flicker frequency in minimal hepatic encephalopathy
Source: Aliment Pharmacol Ther. 2013 Jan 7;37(5):527–36. doi: 10.1111/apt.12199 (PMC3761188; doi:10.1111/apt.12199)

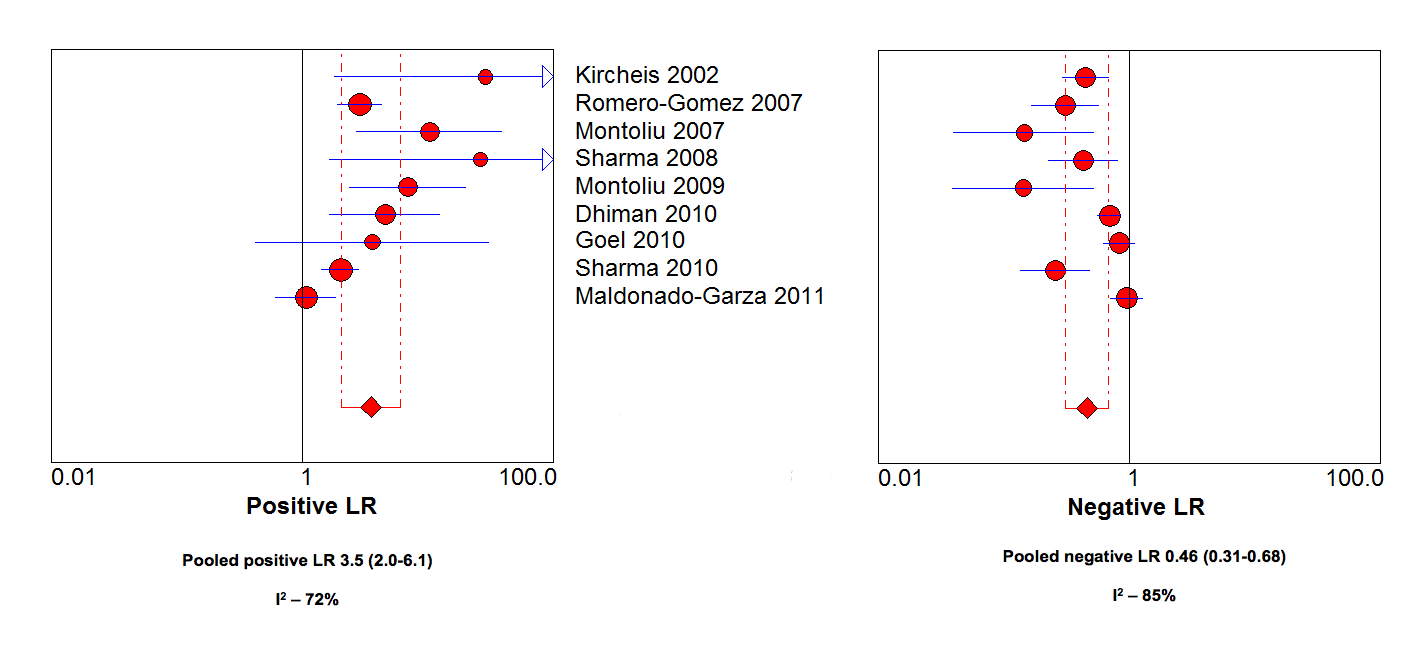

Supplement: Supplementary file 1 [file apt0037-0527-SD1.tif]

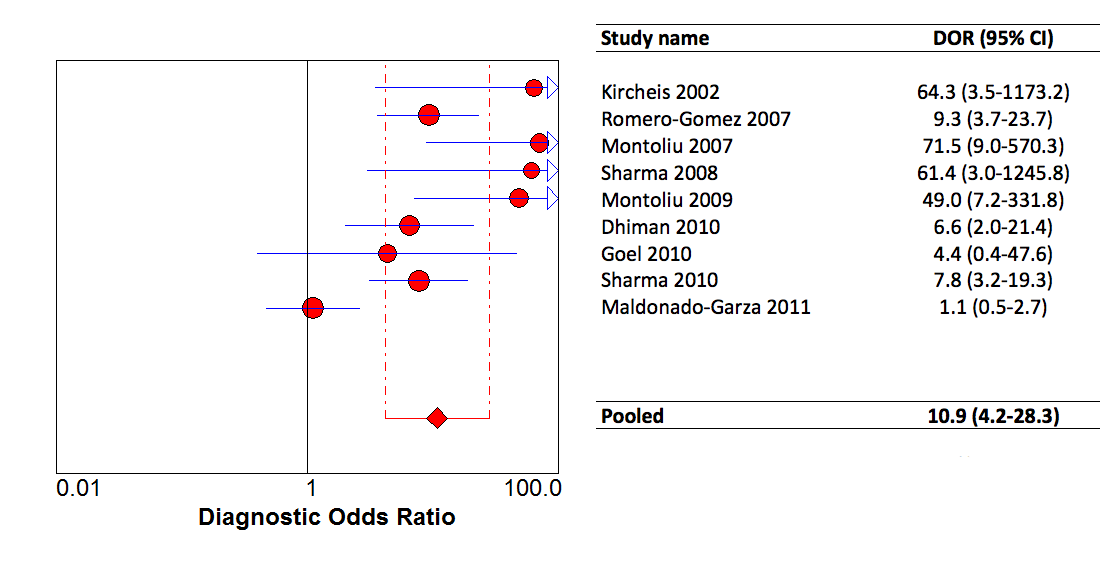

Supplement: Supplementary file 2 [file apt0037-0527-SD2.tif]
